# Intersession Robust Hybrid Brain–Computer Interface: Safe and User-Friendly Approach with LED Activation Mechanism

**DOI:** 10.3390/mi16111264

**Published:** 2025-11-08

**Authors:** Sefa Aydın, Mesut Melek, Levent Gökrem

**Affiliations:** 1 Department of Electronics and Automation, Gumushane University, Gumushane 29100, Turkey; 2 Department of Software Engineering, Gumushane University, Gumushane 29100, Turkey; 3 Department of Electrical and Electronics Engineering, Tokat Gaziosmanpasa University, Tokat 60100, Turkey

**Keywords:** brain–computer interface, classification, correlation alignment, cross-session, Electroencephalography, Electrooculography artefacts, Emotiv Flex, machine learning

## Abstract

This study introduces a hybrid Brain–Computer (BCI) system with a robust and secure activation mechanism between sessions, aiming to minimize the negative effects of visual stimulus-based BCI systems on user eye health. The system is based on the integration of Electroencephalography (EEG) signals and Electrooculography (EOG) artefacts, and includes an LED stimulus operating at a frequency of 7 Hz for safe activation and objects moving in different directions. While the LED functions as an activation switch that reduces visual fatigue caused by traditional visual stimuli, moving objects provide command generation depending on the user’s intention. In order to evaluate the stability of the system against physiological and psychological conditions, data were collected from 15 participants in two different sessions. The Correlation Alignment (CORAL) method was applied to the data to reduce the variance between sessions and to increase stability. A Bootstrap Aggregating algorithm was used in the classification processes, and with the CORAL method, the system accuracy rate was increased from 81.54% to 94.29%. Compared to similar BCI approaches, the proposed system offers a safe activation mechanism that effectively adapts to users’ changing cognitive states throughout the day by reducing visual fatigue, despite using a low number of EEG channels, and demonstrates its practicality and effectiveness by performing on par or superior to other systems in terms of high accuracy and robust stability.

## 1. Introduction

**Figure 1:**
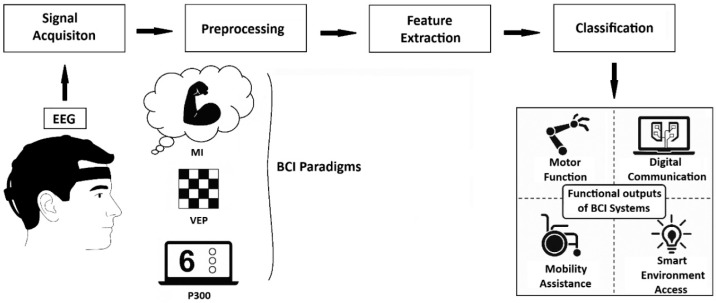
The basic scheme of BCI systems.

Today, Brain–Computer Interface (BCI) systems are attracting intense attention due to their potential to help individuals with severe motor impairments by enabling control of peripheral devices through brain signals. BCI provides a communication or movement channel for individuals who have lost voluntary muscle control by translating brain signals into control commands. These systems aim to increase autonomy and quality of life by allowing individuals to perform tasks such as letter selection, device control, or wheelchair use with their brain activities [1]. In Electroencephalography (EEG)-based BCI studies, several widely adopted paradigms are employed during signal acquisition, including Visual Evoked Potentials (VEPs) [2], Motor Imagery (MI) [3], and P300 [4] responses. The P300 paradigm is characterized by a positive peak in the EEG signal that occurs approximately 300 to 600 milliseconds following the presentation of a task-relevant stimulus, such as a flashing light, a sound, or a specific visual cue. This component is typically elicited when the user consciously recognizes a stimulus, and is easily detectable through non-invasive EEG recordings. In contrast, the MI paradigm relies on the mental rehearsal of motor actions rather than actual physical movement. When a person imagines a specific motion like moving a hand or foot, distinct brainwave patterns are produced, which can be recorded and analyzed using EEG systems. These imagined movements generate neural signals in motor-related cortical areas, making MI a powerful technique for voluntary control in BCIs [5]. The VEP approach, on the other hand, involves presenting users with rhythmic visual stimuli such as blinking lights or oscillating patterns at specific frequencies. These stimuli induce synchronized voltage changes in the visual cortex [6]. When the stimulation frequency exceeds approximately 6 Hz, the response transitions into a Steady-State Visual Evoked Potential (SSVEP), where the brain’s electrical activity synchronizes with the frequency of the stimulus. SSVEP allows for rapid and reliable classification of user intent based on frequency-locked neural responses [7]. All of these paradigms generate EEG data that must be further processed through signal processing algorithms, enabling the system to classify and interpret user intentions effectively. Each paradigm has unique advantages and is selected based on application context, user capability, and system requirements. The basic BCI schematic is shown in Figure 1.

Although BCI systems were developed to benefit people with motor impairments, the paradigms used in the systems create cognitive load on the user and may experience control lapses due to the variable nature of EEG signals and sensitivity to interference [8]. Hybrid BCI systems developed to overcome these limitations aim to increase performance, reliability, and availability by combining multiple signal sources [9]. While EEG is powerful in detecting mental intention, Electrooculography (EOG) offers fast and precise control with its high signal-to-noise ratio. The combination of these two signals provides an interaction that is both voluntary and natural. It has been reported in the literature that hybrid systems offer higher accuracy and flexibility than single BCI systems [10,11]. In particular, integrating EOG into the system increases resistance to signal interruptions and provides intuitive use with minimal training [12]. Thus, EEG–EOG-based hybrid BCI systems are promising for real-world applications by providing faster, more accurate, and more user-friendly solutions [13].

Eye movements, particularly within electrodes placed over the frontal cortex, frequently create EOG artefacts in EEG recordings. These artefacts are considered non-neural interferences that can distort the interpretation of brain activity and potentially be misclassified as unwanted commands in BCI systems [14]. In applications where such an effect is undesirable, various filtering techniques are applied to suppress EOG components. However, in cases where eye activity is consciously monitored [15], these artefacts can be reused as informative signals. Movements such as blinks, vertical and horizontal gaze shifts, and eye closures can be detected and distinguished from EEG data. Once identified, these signals can be used as control inputs for applications such as BCI-based typing systems [16].

Visual stimulus-based BCI systems such as SSVEP and P300 have significant limitations in terms of usability, comfort, and eye health. Since these systems require focusing on flickering Light-Emitting Diodes (LEDs) for a long time, they can cause complaints such as eye fatigue, visual exhaustion, and headaches [17,18]. Visual stimuli that flicker at certain frequencies used in visual stimulus-based systems have the potential to pose risks to eye health in individuals with light sensitivity. High-contrast stimuli, especially in the 15–25 Hz range, increase the risk of epileptic seizures in photosensitive individuals. Moreover, some people are sensitive even to single flashes or frequencies as high as 65 Hz, which raises concerns about the safety of such systems for certain user groups [19]. It has also been reported that repetitive stimuli cause dry eyes and loss of attention [20]. In traditional designs, emphasis is placed on system performance and Information Transfer Rate (ITR), while user experience remains in the background. In conclusion, although visual BCI systems based on vibratory stimuli are effective in principle, their long-term use may be uncomfortable or unhealthy for users [21]. This clearly demonstrates the need for more comfortable stimulus approaches.

Another important challenge faced by BCI systems is that system performance varies between sessions. Factors such as the unstable nature of EEG signals, repositioning of electrodes, impedance change, user fatigue, or distraction change signal patterns between sessions. Therefore, a classifier trained on data obtained in one session may exhibit lower performance in subsequent sessions. This reduces the usability and reliability of the BCI system and, in practical terms, means that users must recalibrate before each use. Calibrating at each session is both laborious and makes continuous use difficult. From a user perspective, BCI behaviour is unpredictable from one session to the next, harming trust and usability. It is not acceptable for independent use if a system that works very well early in the day works poorly when used again later in the day [22]. Addressing variability across sessions and even across individuals is critical to moving any designed BCI system from laboratory settings to everyday life.

The BCI problems that this study focuses on are summarized as follows:

- Flickering stimuli: Visual stimulus-based BCI systems may cause discomfort in the user such as eye fatigue, headache, and risk of epilepsy due to flickering visual stimuli [17]. This limits long-term use of the system, reduces the comfort level, and causes some users to be excluded for security reasons.
- Intersession instability: Intersession variability that occurs due to the unstable nature of EEG signals negatively affects system performance, and the need for recalibration at each session reduces the usability of the system [22]. This situation significantly limits the use of BCI systems in real life.
- Reliability and accuracy: Single BCI systems are not reliable in terms of system security due to their dependence on EEG signals that vary frequently between sessions. EEG alone can experience control lapses due to its variable nature and sensitivity to interference [8]. This situation shows that there is a need for the existence of multi-source hybrid BCI systems.
- System control and security: In BCI systems, activation is critical to prevent unintentional commands and safely initiate user control. Especially in real-world applications, it is important for user experience that not every signal is perceived as a command [13]. It is important to prevent random eye/muscle movements from being perceived as wrong commands in designed systems.

In this study, a two-stage hybrid BCI system was developed in which SSVEP and EOG artefacts were used together. The user is presented with a 7 Hz frequency LED and objects moving in four different directions on a single screen. The system is structured in two stages to ensure conscious activation. In the first stage, it was detected whether the user produced an SSVEP response via the LED by looking at the screen. At this stage, the LED functioned as a “brain-controlled safety switch”. When the LED was detected, the second stage of the system was activated, thus greatly reducing the risk of incorrect commands. The 7 Hz frequency was strategically chosen to reduce the risk of triggering in photosensitive individuals and ensure adequate SSVEP production. In the second stage of the system, the trajectory of the moving object that the user was looking at was determined using EOG artefacts evident in the frontal lobe. Thus, the activation intention was detected via EEG, and the system output command intention was detected via EOG. In the first stage, in frequency domain proportioned trapezoidal features were extracted using Power Spectral Density (PSD). Feature data was classified by Random Forest (RF), Support Vector Machine (SVM), and Bootstrap Aggregating (Bagging) algorithms and accuracy rates of 98.67%, 98.63%, and 99.12% were obtained, respectively. In the second classification stage, only samples with correct LED activation were evaluated; time domain-based power, energy, and 20th degree polynomial features were extracted from these signals. The feature data obtained in the time domain was classified with RF, SVM, and Bagging algorithms, and average accuracy rates of 79.87%, 76.31%, and 81.54% were obtained, respectively. Then, the Correlation Alignment (CORAL) method was applied to the feature data in order to reduce the distribution differences between sessions. CORAL ensures statistical fit between source and target data by aligning covariance matrices [23]. As a result of the classification made with CORAL, the Bagging algorithm increased from 81.54% average accuracy rate to 94.29% accuracy rate, providing the best performance between sessions despite individual variations.

The main contributions that the designed hybrid BCI system aims to provide are presented below.

- Visual comfort: The only vibrating stimulus used in the system is 7 Hz LED, and this frequency value is outside the high risk 15–25 Hz range [19] and is a partially ideal value. All other stimulators in the system are motion-based and do not require vibration. This largely eliminates the visual fatigue problem caused by visual stimulus-based systems in users.
- Intersession stability: In the designed system, the dataset was recorded in two different sessions. By applying the CORAL method to the recorded data, differences between sessions were minimized and the system was aimed to have a stable structure between sessions.
- Reliability and accuracy: The designed BCI system benefits from the high signal clarity of EOG, as opposed to the unstable structure of EEG signals. By processing eye movements with high precision, complex orbits can be classified accurately by the system. The overall effect is a hybrid BCI system that is both user-friendly and reliable.
- System security and activation: In the designed hybrid system, the fact that SSVEP and EOG require approval together via 7 Hz LED provides a natural security control. Since the system switches to control mode only after the safe activation of the first stage has occurred, the user can freely look or blink while the LED is not active; there is no risk of making an unintentional choice.

In this study, it was aimed to provide robustness against intersession performance changes in order to increase the adaptability and generalizability of the previously proposed hybrid BCI system [24] to real-world conditions. The designed system was tested in two different time periods (morning and evening), and the CORAL-based domain adaptation method was used. In this context, the need for recalibration of the system was reduced and not only the instantaneous accuracy rates but also the time-varying cognitive and physiological states of the users were evaluated. In this respect, a system closer to real-world applications has been presented. In addition, consistent results obtained by increasing the number of participants in the study showed that the system can be adapted to different user profiles. Presenting all visual stimuli on a single screen facilitated the integration of the system into real life and increased user ergonomics. In addition, the system, which was tested using fewer features and data with the same number of channels, has been made suitable for portable applications with low resource requirements. All these elements have revealed that the system can maintain high performance, reliability, and ease of use despite its simplified structure.

## 2. Related Works

In this section, a literature review was conducted on EEG-EOG-based hybrid BCI systems, SSVEP-based BCI systems, the intersession stability of BCI systems, EOG artefacts occurring in EEG signals, and the negative effects of visual stimuli on system users.

Hybrid BCI systems aim to offer greater command capacity, performance, and reliability than single BCI systems by combining multiple biological signals. For example, in one study [25], researchers used the EEG–EOG hybrid system for wheelchair control and added state change commands with eye blinks. In another study [15], researchers developed an asynchronous hybrid BCI combining SSVEP and EOG signals for six-degree-of-freedom robotic arm control. In this system, visual stimuli flashing at 15 different frequencies are obtained from EEG, while the SSVEP interface can be opened and closed by the user blinking three times in a row, thus preventing false triggers and reducing visual fatigue by stopping visual stimuli when not needed. In experiments with fifteen participants, the hybrid system provided an average accuracy of 92.1% and an ITR of 35.98 (bits/min). Similarly, researchers [12] proposed a hybrid BCI that uses EEG and EOG signals to control the integrated wheelchair and robotic arm system. Experiments with twenty-two volunteers showed that the MI + EOG hybrid approach can provide sufficient accuracy to control multiple devices in an integrated manner. In another study [26], researchers used a combination of SSVEP, EOG, eye tracking, and force feedback to control a virtual industrial robot arm. In this way, they achieved more precise positioning and object alignment than was possible with EEG. In a different study [27], researchers adapted hybrid methods to the typing interface, developing a BCI that combines SSVEP with a paradigm of letter selection and subsequent eye movement confirmation. Using the hybrid printer system, they achieved an average accuracy rate of 94.8% and an ITR of 108.6 (bits/min) with ten healthy subjects.

Although eye movement signals recorded with EEG are generally seen as artefacts, voluntarily produced EOG signals can be turned into a direct control tool thanks to their strong and repeatable structure. Eyeball movements and electrical potentials produced by the eyelid muscles, independent of the brain, can be easily measured with a few electrodes placed on the forehead and around the eyes. In a study [28], researchers controlled an assistive robotic arm using eye artefacts. In this study, eye blink and eye shift signals, which appear as noise in the EEG, were detected with special algorithms and converted into commands. With this method, tested with five participants, users successfully controlled a robot via the interface using only eye movements. In another study [29], researchers developed a wearable interface with six EOG electrodes to control a game in a virtual reality environment with eye movements. In this system, the user was able to perform seven different eye movement commands. The fact that EOG signals can be classified with such high discrimination reveals that eye movements are information-carrying signals, not noise. In a different study [30], researchers included EOG channels as auxiliary features when classifying EEG signals and achieved a significant performance increase compared to EEG alone. In the study, they achieved 83% accuracy with 3 EEG and 3 EOG channels, approaching the classical 22-channel EEG classification. This result demonstrates that eye signals traditionally considered artefacts are useful biomarkers if evaluated appropriately.

SSVEP-based BCI systems are widely investigated, especially due to their high ITR and short calibration time requirement. SSVEP is a continuous rhythmic EEG response created in the occipital cortex by visual stimuli that repeat at a certain frequency. The user can choose to focus on one of many targets flashing at different frequencies; thus, multiple command candidates can be presented at the same time. For example, in one study [27], researchers developed a high-speed virtual keyboard with 20 different SSVEP targets in a hybrid system. In another study [31], the recognition performance of the bandpass CCA approach proposed by the researchers was increased by subjecting the fundamental and harmonic frequency components of the SSVEP response to correlation analysis in separate sub-bands. This method has increased the reliability of SSVEP-based systems by providing more accurate detection, especially in noisy environments or high-frequency weak SSVEP signals. In the literature, ITRs over 100.0 (bits/min) have been reported to be achieved with SSVEP interfaces containing more than 40 targets [32].

Although flicker-based BCI systems such as SSVEP successful in terms of performance, they are inadequate in terms of user comfort in long-term use. High-contrast and low-frequency flashing visual stimuli can cause eye fatigue, irritation, headaches, and even epilepsy in users after a while. Seeking a solution to these problems, studies have been conducted on the perception of high-frequency stimuli as continuous light in the human eye. However, a study [33] reported that the use of high frequency negatively affects classification performance by reducing the SSVEP signal amplitude. In a hybrid BCI study [15], the screen can completely turn off flicker stimuli when the user does not want them. In this way, the user is exposed to flashing stimuli only when they want to give a command and have the opportunity to rest in between. Results showed that this on-demand stimulation feature significantly reduced visual fatigue and prevented accidental commands. While some studies [33] show that the use of low-contrast flicker is promising, other studies [34] aim to achieve similar performance with motion-based paradigms that can completely replace flicker.

As an alternative to flicker stimuli, BCI paradigms targeting the brain’s continuous visual tracking mechanisms have been developed using moving visual stimuli. The frequency of the stimulus creates a continuous potential in the visual areas of the brain, just like SSVEP. In a study [34], a system with eight targets was designed using visual targets that move radially in growth and contraction. Growing and shrinking moving stimuli were compared with classical flicker stimuli. The results showed that the radial motion paradigm achieved a very high performance with an average accuracy rate of 93.4% and an ITR of 42.5 (bits/min). Participants reported that following constantly growing and shrinking circles with their eyes was much less tiring than flashing lights. In another study [35], different types of moving stimuli were compared and it was observed that there were no significant differences between them in terms of comfort or performance. A different study [36] reported that classical flicker stimuli produced a stronger SSVEP response but lower visual comfort than pattern reversal stimuli. According to a review [37], several studies in the literature have achieved accuracies of 90.0% and above with combinations such as a rotating object flickering at the same time. Another approach to moving stimuli is eye tracking-based selection paradigms. Researchers [38] demonstrated this approach in the smart watch interface, providing a hands-free interaction that does not require calibration. The biggest advantage of these interfaces is that the system generates commands only when the user consciously follows a goal, and the system does not react to random glances. In addition, it is comfortable for many users as natural eye movement is sufficient with low cognitive load. In some hybrid BCIs, flicker-triggered SSVEP is combined and then confirmation with eye movement is used. Thus, a multi-stage selection was made using both brain and eye signals.

Practical use of a BCI system depends on its consistent performance over multiple sessions. EEG signals vary between sessions for many reasons, such as electrode position changes, skin-resistance differences, and the mental/emotional state of the user. This situation creates the need for recalibration before each new use, making the use of BCI troublesome. In one study, researchers [39] discussed machine learning-based transfer learning methods and neurophysiological variability predictors, presenting a review to eliminate these variations that reduce performance in BCI systems. In their review, they stated that approaches that extract common features between datasets to reduce the need for calibration promise success. As a matter of fact, in another study [40], a transfer learning algorithm was developed that aims to shorten the training time at the beginning of each session. In the evaluation made with 18 sessions of data from 11 stroke patients, it was shown that training the model with the transfer of previous sessions provided an accuracy increase of over 4% compared to pure new session training. Especially in sessions where the performance was below 60% in the first calibration, this transfer method provided an additional 10% improvement, making it possible for more patients to benefit significantly from BCI.

## 3. Materials and Methods

### 3.1. EEG Device

Studies show that Emotiv (EMOTIV Inc., San Francisco, CA, USA), Quik-Cap (Compumedics Neuroscan, Abbotsford, Victoria, Australia), and MindWave (NeuroSky Inc., San Jose, CA, USA) EEG devices are frequently used, and among these, Emotiv (EMOTIV Inc., San Francisco, CA, USA) is frequently preferred due to its low cost, sufficient number of channels, and ease of use. The Emotiv Flex EEG device used in the study has 32 channels and 2 reference electrodes, and the electrode positions comply with the international 10/20 system. Offering approximately 9 h of uninterrupted use with its wireless structure and rechargeable battery, the device provides 128–256 Hz sampling rate and 16–32 bit resolution values [41].

### 3.2. Participants

This study was conducted with the participation of 15 healthy subjects (9 males and 6 females) between the ages of 20 and 30 (avg. 22.5), without any addiction or chronic disease. The participants were informed in detail about the study and were included in the study by filling out the informed consent form. The experiments were conducted with the approval of the ethics committee of Trabzon Kanuni Education and Research Hospital numbered 23618724.

### 3.3. Normalization

Normalization is a fundamental pre-processing step that directly affects model performance by making data at different scales comparable [42]. In this study, Z-score normalization was used in order to reduce the amplitude and signal-to-noise ratio (SNR) differences that may occur between two sessions. Z-score is a standardization method that expresses the distance of each data point from the mean in its distribution in terms of standard deviation and is calculated with Equation (1).

(1) $$z=\frac{x-\mu }{\sigma }\;$$

In Equation (1), x represents the data point, μ represents the mean of the cluster, and σ represents the standard deviation [43].

### 3.4. Correlation Alignment (CORAL)

Distribution differences between biological signals recorded in different sessions may negatively affect the generalization success of machine learning-based classifiers. In this study, the CORAL method was used to reduce this problem. CORAL provides statistical fit without the need for label information by aligning the covariance matrices of the source and target datasets. This method, which is based on whitening the source data and rescaling it according to the target distribution, improves transfer performance by increasing intersession compatibility [23].

### 3.5. Power Spectral Density (PSD)

In the first classification stage of the study, where 7 Hz LED was detected using SSVEP, the PSD method was used to analyze the frequency components of EEG signals. With PSD, EEG data, which is difficult to analyze in the time domain, is made more meaningful by displaying the power distribution of the signal on the frequency axis. The Welch method was preferred because it provides low variance and stable results. The Welch method works by dividing the data into K sub-segments of equal length that partially overlap each other. The PSD estimate is expressed by Equation (2).

(2) $$P_{welch}(f)=\frac{1}{K.L.U}\sum_{i=0}^{K-1}{\left|\sum_{n=0}^{L-1}x_{i}\left(n\right).\omega \left(n\right)e^{-j2\pi fn}\right|}^{2}$$

In Equation (2), K refers to how many segments the data is divided into, L is the length of each divided segment, $\omega \left(n\right)$ is the window function, and $x_{i}\left(n\right)$ is the nth sample of the i. segment. U represents the strength of the windowing function [44].

### 3.6. Numerical Integration (Trapezoidal)

The trapezoidal method, one of the numerical integration methods, was used to approximately calculate the area under the PSD values in the 4–10 Hz and 6–8 Hz frequency ranges for the detection of 7 Hz LED. The trapezoidal method calculates the area under the function by approximating the positive function f to be integrated with piecewise linear curves in the range [*a*, *b*]. The trapezoidal method is frequently preferred in power analysis of biological signals such as EEG/EOG due to its ease of application and low computational cost, as well as providing sufficient accuracy. Using the trapezoidal method, the area under a function curve is calculated with Equation (3).

(3) $$\int_{a}^{b}f\left(x\right)dx=\frac{h}{2}\left[f\left(x_{0}\right)+2\sum_{i=1}^{n-1}f\left(x_{i}\right)+f(x_{n})\right]$$

In Equation (3), the parameters a and b represent the integration limits of the f function, ${(x}_{0},x_{1},\ldots ,x_{n})$ represent the equal interval points where the integration limit is divided, and h represents the width of the lower trapezoids [45,46].

### 3.7. Polynomial Curve Fitting Method

In this study, the polynomial curve fitting method was used to classify EOG artefacts in the time domain. This method is the process of creating a polynomial model based on the least squares method on the dataset. The most appropriate polynomial coefficients are obtained by minimizing the total error between the start and end data points of the function and the model [47]. The $y=P_{1}(x)$ polynomial model fitted for y=f(x) with starting point $x_{0}$ and end point $x_{1}$ is expressed by Equation (4).

(4) $$p\left(x\right)=a_{0}x^{n}+a_{1}x^{n-1}+\cdots +a_{n-1}x+a_{n}\;$$

In Equation (4), n represents the degree of the polynomial and $a_{i}$ represents the polynomial coefficients. In the study, the polyfit function was used to fit a polynomial curve to certain data points.

### 3.8. Data Acquisition

**Figure 2:**
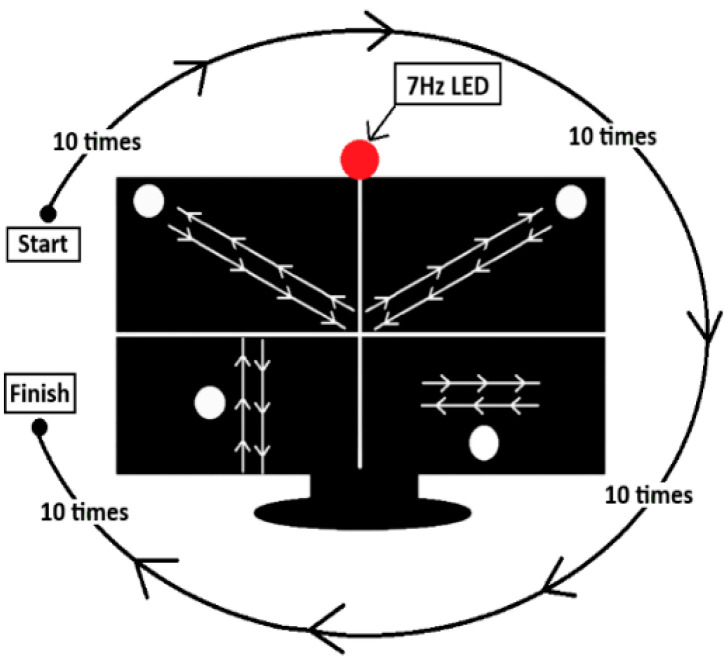
Experimental protocol and task order of movement trajectories applied in this study.

In the data acquisition, an Emotiv Flex EEG device, a laptop with an Intel Core i7–12650 processor, and a 31.5-inch LCD screen with a 260 Hz refresh rate were used as hardware infrastructure. For visual stimulation, an LED flashing at a frequency of 7 Hz was placed at the upper middle point of the screen, and the subject was positioned on a chair approximately 90 cm away from the screen. The procedure was explained to each subject in detail before the experiment, and their consent was obtained. The orbital task order that the subjects were asked to follow is shown in Figure 2.

The subjects were fitted with an EEG cap using saline water. The contact level of the electrodes was verified via EmotivPRO software (v3.5.3), aiming for at least 98% signal accuracy. Electrode placement was made in accordance with the international 10–20 system, and contact quality was optimized with saline solution before each session. In the study, only four EEG channels (Fp1, F7, F8, and Fp2) in the frontal lobe region were actively used. This channel selection was made both to reduce system complexity and target regions where artefacts associated with eye movements can be obtained at the highest level [48]. Data recording in the study was carried out in two sessions.

#### 3.8.1. Session 1—Morning Trials

**Figure 3:**
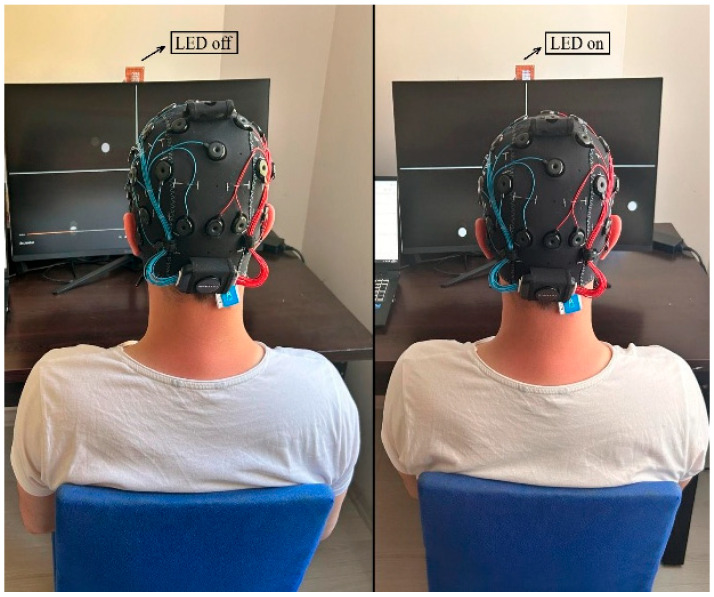
Experimental setup during the signal recording phase, showing EEG device attachment, subject posture, and LED stimulus configuration.

The first session was held to record the cognitive performance differences of users in the morning hours (08:00–10:00). The session started with activating the 7 Hz LED. The experiment was started with a 3 s initial warning sound. Moving balls were activated, and the participants were first asked to follow the left-cross motion trajectory moving in the upper left corner of the screen for 10 s. The experiment ended with a 3 s final warning sound. The visual of the data recording stage is shown in Figure 3.

**Figure 4:**
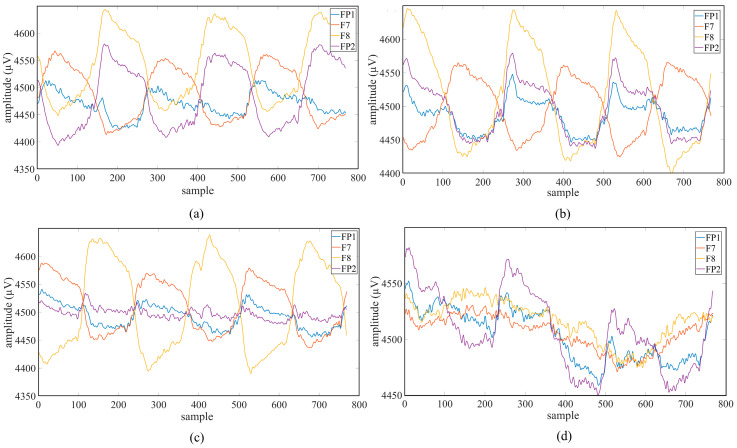
Average value graphs of Session 1: (**a**) left-cross movement trajectory; (**b**) right-cross movement trajectory; (**c**) right–left movement trajectory; (**d**) up and down movement trajectory.

A total of 16 s of data were recorded while the subjects watched the target with their eyes. The same protocol was repeated for other movement trajectories, and 10 repetitions were performed for each class. After the LED was turned off, recordings were taken for the passive viewing class representing random eye movements. Subjects were asked to perform random gazes without focusing on any target. A total of 50 records were obtained from each subject with 10 repetitions for 5 different classes. The average value graphs of the movement trajectory data of the first session obtained from a randomly selected subject (Subject-2) are shown in Figure 4.

#### 3.8.2. Session 2—Evening Trials

**Figure 5:**
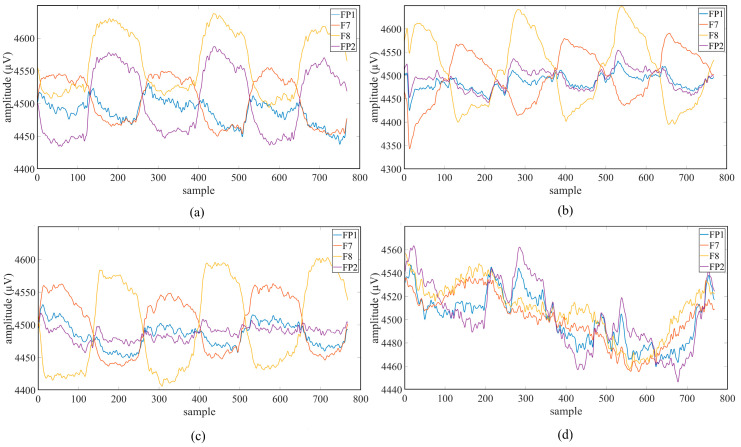
Average value graphs of Session 2: (**a**) left-cross movement trajectory; (**b**) right-cross movement trajectory; (**c**) right–left movement trajectory; (**d**) up and down movement trajectory.

The second session was held in the evening (16:00–18:00) in order to evaluate the stability of the system against the individuals’ cognitive and neurophysiological changes during the day. The EEG head was repositioned, preserving the channel placements and impedance levels used in the first session, and the electrodes were contacted with saline (salt) water again. To avoid signal differences between sessions, special care was taken to place the EEG electrodes in exactly the same positions. The data collection procedure was repeated, remaining exactly the same as the first session, and 50 records were obtained with 10 repetitions for 5 different classes. The average value graphs of the movement trajectory data of the second session obtained from a randomly selected subject (Subject-2) are shown in Figure 5.

The signals obtained in each session were recorded with a sampling frequency of 256 Hz, and the raw data was stored in “.csv” format. A total of 1500 records were obtained from all subjects. Data were transferred to Matlab (R2023b) environment for signal processing stages.

## 4. Results

**Figure 6:**
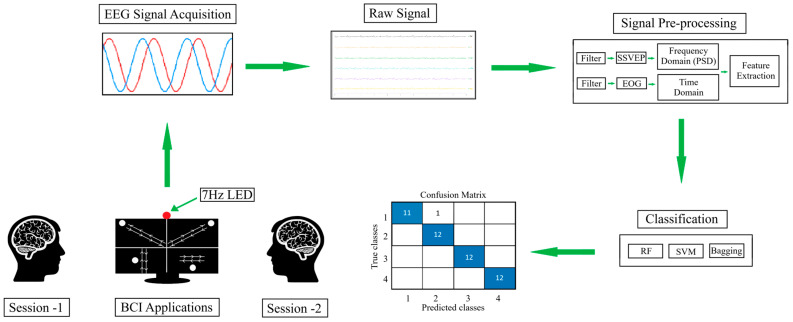
Scheme of the proposed hybrid BCI system illustrating the signal acquisition, processing, and classification stages.

The dataset created using the proposed approach consists of five classes. Four of these classes were recorded while users followed visually guided moving object trajectories while the LED flickering at a frequency of 7 Hz was active. The fifth class was obtained when the LED was off and the user performed random eye movements. This structure enabled the system to be constructed in a two-stage classification structure. The scheme of the designed hybrid BCI system is shown in Figure 6.

In the first stage, LED on (SSVEP present) and LED off (SSVEP absent) states were distinguished. In the second stage, the data of four different moving trajectories recorded only when the LED was on were classified. The stability of the system between sessions was tested by using all the data collected in the morning session as training data and all the data in the evening session as test data. In each session, a total of 16 s of data (4096 samples × 4 channels) were recorded, with 3 s audio warnings at the beginning and end.

**Figure 7:**
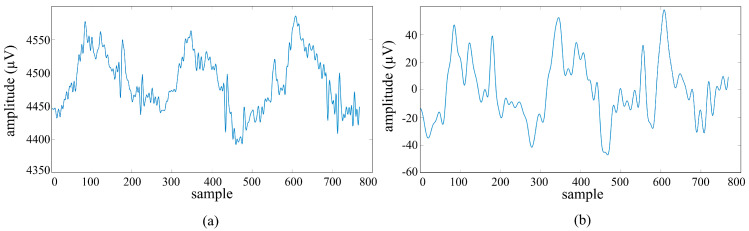
Acquired from the fp1 channel: (**a**) unfiltered signal; (**b**) signal filtered with a 1–15 Hz 5th-order Butterworth bandpass filter.

In order to prevent the system from being affected by the beginning and ending warning sounds, these sections were removed from the signal and only signals of 10 s (2560 samples × 4 channels) were evaluated. Then, these raw signals were divided into 3 s segments with 1 s overlap, and five 3 s signals (768 samples × 4 channels) were obtained from each trial. Signals were passed through a 5th order Butterworth bandpass filter in the range of 1–15 Hz via the Matlab function filtfilt command. Butterworth filters are frequently preferred in EEG processing applications because they provide a flat frequency response in the passband and preserve the amplitude components of the signal [49]. Filters of different orders were tested, and the optimal performance was achieved in the 5th-order filter, considering the balance between passband sharpness and signal distortion. An example signals of the Fp1 channel before (Figure 7a) and after (Figure 7b) filtering is shown in Figure 7.

**Figure 8:**
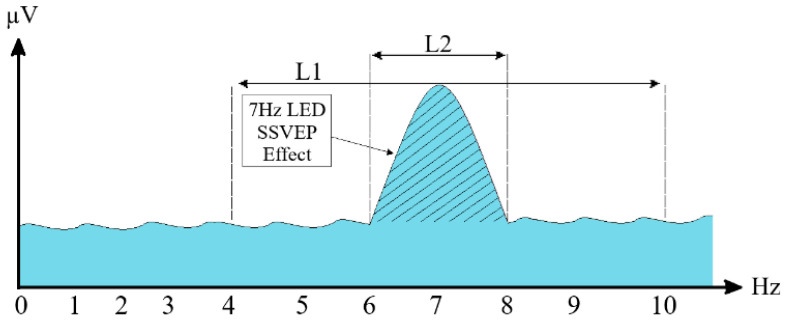
SSVEP potentials occurring in the LED on position.

Filtered signals were converted from time domain to frequency domain using the Welch PSD method [44]. Using the Hamming window, windowing was performed with a length of 640 samples and an overlap of 639 samples. Hamming windowing parameters used in PSD analysis were determined by experimental methods in accordance with the data structure. It is stated in the literature that the correct selection of window length and overlap ratio plays an important role in establishing a balance between frequency resolution and temporal sensitivity [50,51]. Frequency ranges of 4–10 Hz and 6–8 Hz were determined on the spectrum obtained from PSD, and the areas of these two frequency bands were calculated with the trapezoidal integration method [52], which is frequently used in the literature to calculate the band power. The calculated values are compared to each other. Using the obtained proportional trapezoidal features, it was aimed to detect the SSVEP activity generated using 7 Hz LED. The SSVEP created when the LED is on is represented in Figure 8.

In Figure 8, while the L1 length represents the SSVEP response occurring in the 4–10 Hz range, the L2 length represents the SSVEP response occurring in the 6–8 Hz range. By proportioning these two lengths to each other, a proportional trapezoidal feature was obtained. Z-score normalization was applied to the obtained feature data in order to balance inter-individual and intersession amplitude changes, making the mean 0 and the standard deviation 1 [53]. Normalized feature data was classified using RF, SVM, and Bagging algorithms. The SVM algorithm was configured using the fitcecoc function in the Matlab environment. With the Bagging algorithm implemented through the fitcensemble function, it aimed to increase classification performance by training more than one weak learner on data subsets. The RF algorithm was implemented using the Matlab function TreeBagger. The classification accuracy rates obtained are presented in Table 1. In the table, Class-1 represents the moving trajectory data recorded in the LED active position and Class-2 represents the random gaze data recorded in the LED off position.

**Figure 9:**
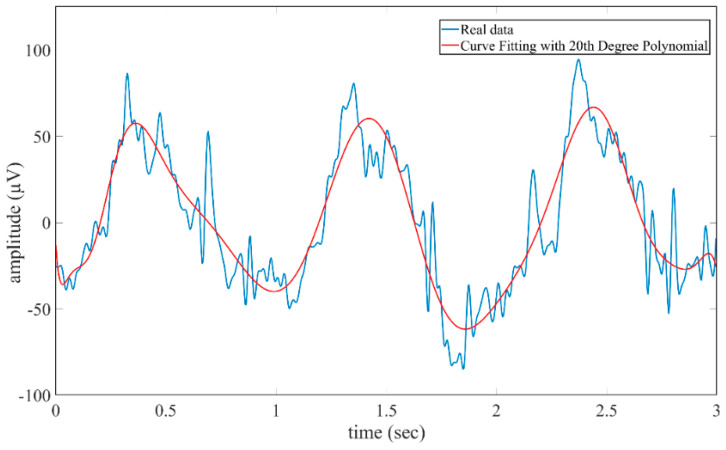
Polynomial curve fitted using 20th-degree polynomial for the signal of Fp1 channel.

When Table 1 is examined, it is seen that the Bagging learning algorithm exhibits the best performance for the first classification stage with an accuracy rate of 99.12%. The second classification stage was carried out on the dataset created using the raw forms of the data correctly classified by the algorithms in the first stage. At this stage, a 4th-degree Butterworth filter, which has the ability to filter the signals without distorting the amplitude components of the signal thanks to its flat frequency response in the pass band [49], was applied to the signals in the range of 0.5–32 Hz. After the filtering process, signal power, signal energy, and polynomial fitting feature extraction methods based on time domains were used. The polynomial fitting method was extended to the 20th degree and all obtained polynomial coefficients were included in the feature vector. Higher-order polynomials are effective in improving classification performance by representing the structural trends of the signal in more detail [47]. Figure 9 shows the curve created using the right-cross motion trajectory and fitted using the 20th-degree polynomial for the signal of the Fp1 channel.

**Figure 10:**
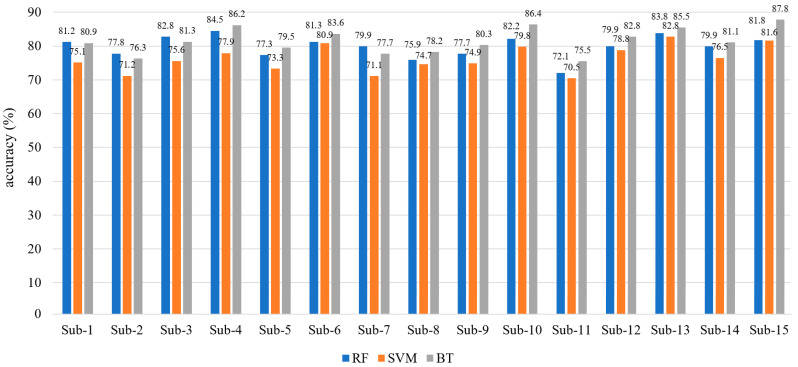
Average accuracy rates of the second classification stage (without CORAL applied).

The obtained feature data was applied to RF algorithm was implemented via the TreeBagger function, and 50 decision trees were used in the model. The number of trees was fixed at the point where the accuracy performance remained stable within the confidence interval (95%) in the preliminary tests. The feature subsets for each tree were randomly selected, which aimed to reduce the risk of over-learning of the model. The obtained feature data were classified with a multi-class SVM model using the fitcecoc function in the Matlab environment. The SVM hyperparameters were automatically adjusted with the Bayesian optimization method, and the combination that provided the best cross-validation performance was selected. The Bagging algorithm was structured as an ensemble model consisting of 100 decision trees, each with a maximum depth of 15, using the fitcensemble function. The number and depth of trees were determined in the preliminary tests to provide the optimum balance between model complexity and processing time. The average accuracy rates of classifications performed over 10 independent repetitions are given in Figure 10.

Figure 10 shows the average accuracy rates of the second classification stage. Average accuracy rates of 79.87%, 76.31%, and 81.54% were obtained for the RF, SVM, and Bagging algorithms, respectively. It can be observed that the Bagging algorithm provides better performance than other algorithms.

**Figure 11:**
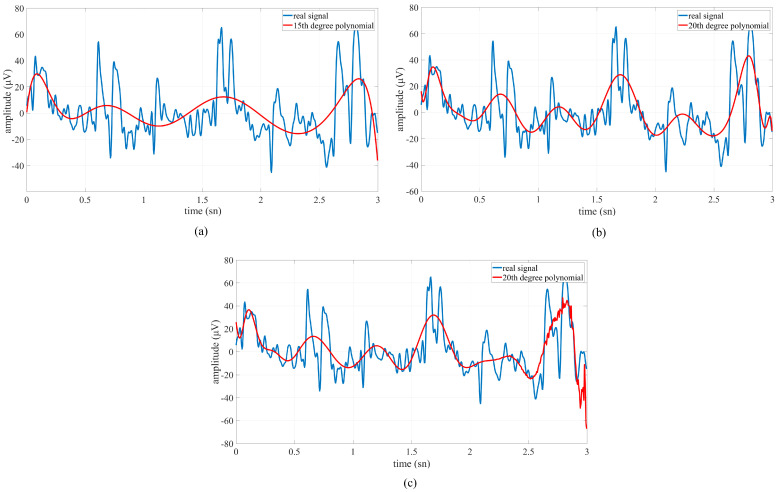
Comparison of polynomial fitting curves with the original signal. (**a**) The 15th-degree polynomial captures the general low-frequency trend of the signal while tracking short-term fluctuations to a limited extent. (**b**) The 20th-degree polynomial exhibits a better fit by compensating for both the slower trend and the local task-related variations in the signal; this degree was considered a candidate to optimize the generalization-fitting balance. (**c**) The 25th-degree polynomial tends to overfit local fluctuations, especially in the extreme regions of the window and at sharp change points, indicating a potential risk of overfitting.

In signal processing applications, overfitting is the situation where the model learns the patterns in the training data in too much detail and fails to capture the general structure underlying these patterns. Higher-order polynomials can pose a risk of overfitting, especially in small datasets. Therefore, the 20th-order polynomial used in this study was systematically tested for its impact on classification performance. Figure 11 compares the approximations obtained with the 15th-, 20th-, and 25th-order polynomials with the original signal.

While the lower-order (15th) polynomials reflect the underlying signal trend, they are found to underrepresent high-frequency components. On the other hand, the 25th-order polynomial overfits local fluctuations, suggesting that it overfits noise components rather than the physiological characteristics of the signal. To quantitatively demonstrate the performance impact of the selection of the polynomial degree, features obtained for three different degrees (15, 20, and 25) were classified using the Bagging classifier. The accuracy rates of the classification process are given in Table 2. In Table 2, Class-1 represents the left-cross movement trajectory, Class-2 represents the right-cross movement trajectory, Class-3 represents the right–left movement trajectory, Class-4 represents the up and down movement trajectory, and n represents the polynomial degree.

The results (Table 2) show that the average accuracy rate (81.95%) achieved with the 20th-degree polynomial is higher than both the 15th degree (74.10%) and 25th degree (80.80%). This demonstrates an optimal point where the model complexity is not excessive but rather better represents the meaningful variations in the signal. In particular, the 87.80% accuracy of the 20th-degree polynomial on the Class-4 samples supports the power of this approach in capturing task-relevant components of the signal.

The purpose of the polynomial approach is to represent a slow trend overlaid on short-term fluctuations in the signal. Very low degrees lead to underrepresentation due to high bias, while very high degrees lead to overfitting due to high variance. The selection of the 20th-degree polynomial was based on both this theoretical trade-off and the empirical accuracy increase shown in Table 2. Thus, model performance was systematically tested for overfitting. This additional analysis supports the fact that the 20th-degree polynomial was not chosen randomly but rather through experimental observation and statistical validation. The results demonstrate that physiologically meaningful signal trends can be captured and that classification accuracy is significantly improved with this optimization.

### 4.1. CORAL Adaptation

**Figure 12:**
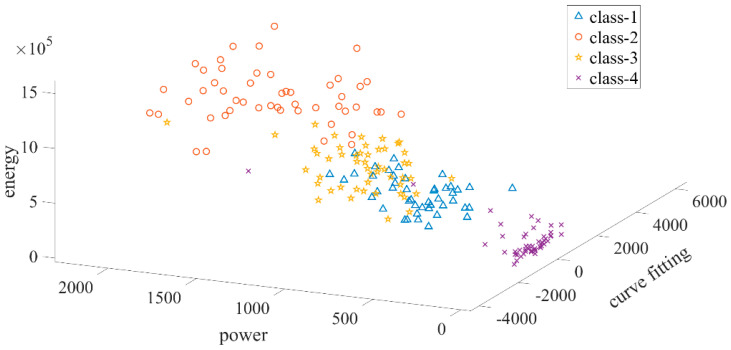
Three-dimensional scatter plot of power, energy, and polynomial curve fitting features of the FP2 channel.

In order to reduce the statistical distribution differences between the two sessions in the study, the CORAL method was applied to the feature data of the second classification stage. CORAL provides cross-domain statistical agreement by aligning the covariance matrices of source (training) and target (test) datasets. Within the scope of this method, the covariance matrix of the normalized training data was whitened and then recolored according to the covariance structure of the test data, making the source data statistically compatible with the target distribution. Thus, the generalization ability of the model across sessions is increased [23]. In order to see the distribution of the obtained features, a scatter plot of the randomly selected FP2 channel is given in Figure 12. In the figure, Class-1 represents the left-cross movement trajectory, Class-2 represents the right-cross movement trajectory, Class-3 represents the right–left movement trajectory, and Class-4 represents the up and down movement trajectory.

As can be inferred from Figure 11, the features can be clearly distinguished. Then, the feature data was classified using RF, SVM, and Bagging machine learning algorithms, as in the first classification stage. All of the data recorded in the first session (early in the day), scaled using the CORAL adaptation method, was used as training data, and all of the data recorded in the second session (late in the day) was used as test data. The RF model was constructed using the TreeBagger function in the Matlab environment and implemented to include a total of 50 decision trees. This number was determined according to typical values suggested in the literature [54,55] and the accuracy–stability balance provided in preliminary experiments. Each tree was trained on randomly selected feature subsets. Model outputs were evaluated by converting from cellular format to numerical form in order to make them suitable for numerical analysis of class labels. The SVM algorithm was constructed with a one-vs-one strategy using the fitcecoc function. Hyperparameter optimization was performed with the Bayesian optimization algorithm. In this process, KernelFunction, KernelScale, and BoxConstraint parameters were optimized; the best model was determined as a result of 30 iterations of the experiment. The Bagging algorithm was implemented using the fitcensemble function, and a model consisting of 100 decision trees, each with a maximum split depth of 15, was constructed in order to reduce the risk of overfitting and increase the generalizability of the model. These parameters were determined by examining previous similar studies [12,56] and experimental accuracy analyses. All classification processes were carried out over 10 independent repetitions and the average accuracy rates, ITR, precision, recall, and F1-measure values obtained are given in Table 3. In Table 3, Class-1 represents the left-cross movement trajectory, Class-2 represents the right-cross movement trajectory, Class-3 represents the right–left movement trajectory, and Class-4 represents the up and down movement trajectory.

When Table 3 is examined, it is seen that the RF algorithm performs well with an average accuracy rate of 93.80%, an ITR value of 37.54 (bits/min), and a precision of 94.07%. The algorithm stands out with its 96.53% accuracy rate, especially in Class-4. The SVM algorithm showed a performance close to RF, with an average accuracy rate of 92.02%, an ITR value of 35.38 (bits/min), and a precision of 92.82%. While a high accuracy rate of 98.66% was achieved in Class-4, a lower performance was achieved compared to other algorithms with an accuracy rate of 87.18% in Class-1. The Bagging method showed the highest overall performance with an average accuracy 94.29%, an ITR of 38.35 (bits/min), and a precision of 94.55%. The algorithm stands out with accuracy rates of 96.84% and 95.00%, especially in Class-2 and Class-3, respectively. Overall, the Bagging algorithm exhibited the best performance with high accuracy rate and ITR. While the RF algorithm showed performance close to Bagging, SVM fell behind the other two algorithms despite achieving high accuracy rates in some classes. The results obtained for this study revealed that the CORAL method is an effective domain adaptation method in classifying EOG artefakt signals found in EEG signals. In addition, the performance of the Bagging algorithm showed that ensemble learning methods were effective for the current study.

### 4.2. Feature Extraction

Given that the 20th-degree polynomial expansion produces a large number of features, and that many of the generated features may be correlated and increase noise, a feature selection step was applied to the dataset to reduce the high dimensionality. A one-way Analysis of Variance (ANOVA) F-test approach was chosen, which evaluates each feature by testing for significant differences in its mean value across classes. This technique effectively scores how well each feature discriminates between classes, helping to identify and remove irrelevant features. The process and outcome stages are outlined below:

- ANOVA test on all features: A one-way ANOVA was applied to all 92 features (23 features × 4 channels) extracted from 4 channels (Fp1, F7, F8, and Fp2) to assess their significance in distinguishing between target classes. The ANOVA examined the inter- and intra-class variance of each feature to determine whether the class means of that feature differed significantly.
- Selection criterion (*p* < 0.05): Using a significance threshold of *p* < 0.05, 66 features that showed statistically significant differences between class means were selected. Features falling outside the threshold were removed from the data. In other words, significant features with *p*-values below 0.05 were retained for the model.
- Retraining with selected features: The classification model was retrained using only the selected features using ANOVA. The average accuracies obtained for the RF, SVM, and Bagging algorithms are close to the performance using all 92 features, and no significant decreases are observed. The RF algorithm decreased from 93.80% to 90.93%, the SVM algorithm from 92.02% to 90.11%, and the Bagging algorithm from 94.29% to 92.09%. The highest accuracy rate of 92.09% was obtained using the Bagging algorithm. All classifiers exhibited slightly lower, but still high, accuracies in the classification results. Despite these modest decreases, the results obtained with the reduced feature sets remained robust and competitive. The classification results showed that the most useful components of the data were preserved.
- Impact on performance and robustness: The accuracy rates obtained with the reduced feature set indicate that the extracted features are relatively meaningless. Removing these less informative features did not significantly degrade model performance. On the contrary, it increased model robustness by removing redundant information. By reducing the feature set, noise that could hinder class identification was also reduced. This additional step validated the robustness of our approach and increased reproducibility by focusing on the most useful features while partially preserving model performance. The resulting accuracy rates are shown in Table 4. In Table 4, Class-1 represents the left-cross movement trajectory, Class-2 represents the right-cross movement trajectory, Class-3 represents the right–left movement trajectory, and Class-4 represents the up and down movement trajectory.

ANOVA-based feature selection reduced the size of the feature set from 92 to 66 while maintaining high classification performance. The classifier achieved 92.09% accuracy rate with this reduced set, demonstrating that the model retained the most informative and discriminative features. This result confirms that the proposed system is not overly sensitive to noisy or correlated inputs. Furthermore, the selection process increased both the interpretability and reproducibility of the model, improving its generalization ability.

### 4.3. Cross-Session Evaluation (Wilcoxon Test)

In this study, EEG data were collected from participants in two separate recording sessions conducted at different times of the day (morning and evening) under consistent experimental conditions. The entire dataset from the morning experiments was used to train the Bagging classifier, while the entire dataset from the evening experiments was used solely to test the trained model. This clear temporal separation between the training and testing phases allows for the assessment of the system’s robustness to non-stationarity and diurnal signal variability, a critical factor for practical BCI deployment.

The Wilcoxon signed-rank test was applied to evaluate the temporal robustness and generalizability of the proposed hybrid BCI system and to examine whether there was a significant difference between the classification accuracies obtained. This test was chosen because of its ability to statistically evaluate the median difference between two paired measurement groups when the normal distribution assumption is not met. A *p* < 0.05 value, widely used in the literature, was used as the threshold for statistical significance in the analysis. The Bagging algorithm, which provides the highest accuracy rate within the scope of the study, was used in the tests. The model was trained with data from the morning session; the accuracy rate for the first dataset (morning) was obtained by testing with morning data, and the accuracy rate for the second dataset (evening) was obtained by testing with data from the evening session. These accuracies were subjected to the Wilcoxon signed-rank test, and the results are presented in Table 5. In Table 5, Class-1 represents the left-cross movement trajectory, Class-2 represents the right-cross movement trajectory, Class-3 represents the right–left movement trajectory, and Class-4 represents the up and down movement trajectory.

The Wilcoxon signed-rank test yielded a *p* = 0.125 result, demonstrating that class-based accuracies did not differ significantly across sessions, demonstrating consistent and stable classification performance across sessions.

Although a slight decrease in accuracy was observed, performance remained consistently high across sessions. A *p* value of 0.125 (*p* > 0.05) indicated that there was no statistically significant difference between training and testing accuracies across sessions. This result demonstrates that the proposed system maintains robust classification performance across recording times, confirming its potential for real-world use where recording variability is unavoidable.

## 5. Discussion and Future Work

Studies in the literature have revealed that hybrid BCI systems created with the integrated use of EEG and EOG artefacts offer significant advantages in terms of accuracy and flexibility [9]. SSVEP-based systems stand out with their high ITR rates; however, they have limiting factors such as user comfort, individual differences, and intersession variability [17,22]. Recently, properly processed EOG artefacts have attracted attention as a low-cost and fast-reacting alternative control interface [12]. Domain adaptation methods, especially for eliminating statistical differences between sessions, have come to the fore in the literature. In addition, it has been observed that systems developed with a low number of channels provide significant advantages to the user in terms of portability, ease of use, and hardware simplicity [30].

In this study, a hybrid BCI system that uses EEG signals and EOG artefacts is proposed to overcome the problems of session variability, visual stimulus-induced disturbances, reliability, and involuntary system activation encountered in traditional EEG-based systems. With the proposed system, a structure that is resistant to the physiological and psychological fluctuations of the users during the day is aimed at. Accordingly, data recordings were carried out in two different sessions, morning and evening. All moving objects in the designed interface are presented to the user simultaneously, aiming to ensure that the system is suitable for use in real-life conditions. The designed system has a two-stage classification structure. In the first stage, by extracting the average trapezoidal features in the frequency domain, SSVEP activation corresponding to 7 Hz LED was detected as a safe trigger mechanism. In the second stage, using raw EOG artefacts in EEG signals, power, energy, 20th degree polynomial coefficients, and time domain features were extracted in order to classify four different movement trajectories. A comprehensive evaluation was conducted to ensure the robustness and generalization of the proposed hybrid BCI system. The analysis of polynomial fitting revealed that model accuracy strongly depended on the polynomial order, with the 20th-degree polynomial providing the optimal balance between bias and variance, achieving the highest overall accuracy (81.95%) without overfitting. Models were trained using Bagging, SVM, and RF algorithms. The second session data was used to test the models. The results revealed that the Bagging algorithm achieved the highest success with 99.12% accuracy in the first classification stage and demonstrated the reliability of the ensemble learning approach for the detection of low-frequency visual stimuli. In the second stage, the data was first classified without applying any adaptation method. Then, the classification process was repeated by applying the CORAL adaptation method and the results were compared. The Bagging algorithm achieved the best performance across sessions, reaching an accuracy rate of 94.29% from an average accuracy rate of 81.54%, despite individual variations. Feature selection was performed to evaluate the extracted features. Feature selection using the ANOVA F test (*p* < 0.05) effectively reduced the feature set from 92 to 66 while maintaining high classification performance. The Bagging classifier achieved 92.09% accuracy with the reduced set, confirming that redundant and noisy features were successfully removed without compromising model robustness. Finally, temporal robustness was verified using the Wilcoxon signed-rank test (*p* = 0.125), which showed no statistically significant difference between morning and evening session accuracies. This confirmed that the proposed system exhibited stable and reliable performance across sessions and its suitability for real-world BCI applications. Basic information about similar studies conducted in the field is given in Table 6.

In the study combining SSVEP and eye movements, researchers [57] reported 81.67% accuracy with their proposed approach. This rate is below the accuracy level of the proposed study and can be attributed to the fact that the Bayesian update mechanism used is not robust enough to individual differences. In another study, researchers [12] presented a system that provides over 80% accuracy by combining MI and EOG, but this system has limitations in practical applications due to both the training requirement and low ITR. Compared to these studies, the proposed system combines the high ITR advantage of SSVEP with the fast and involuntary responses of EOG and offers an intuitive control infrastructure that does not require training. In addition, the double-stage control (SSVEP + EOG approval) offered by our system in terms of security eliminates the risk of users issuing unintentional commands, increasing the level of reliability for real-world applications. With these aspects, the proposed system offers a hybrid BCI approach that prioritizes both user comfort and technical accuracy, and is relatively more balanced, safe, and applicable compared to existing studies in the literature.

Again, when Table 6 is examined, it is seen that high accuracy rates and ITR values are obtained in the developed systems [15,27]. However, these systems require complex processing steps due to the high number of channels and are not sufficient in terms of comfort. In the proposed study, a similar accuracy rate (94.29%) was achieved by using only four channels (FP1, F7, F8, and FP2), and a speed sufficient for practical applications was achieved with an ITR of 38.35 (bits/min). Another notable example is the 21-channel EEG-based system developed by researchers [59], which achieved 98.8% accuracy and 44.1 (bits/min) ITR. However, the equipment that such systems must carry is quite complex and costly. In this context, obtaining high accuracy and sufficient ITR with only four channels and minimal hardware in the proposed study is an important advantage in terms of hardware cost and user ergonomics.

In our study, we aimed to increase the adaptability and generalizability of the hybrid BCI system previously presented by researchers [24] to real-world applications. The proposed BCI system stands out with its resilience to performance losses that occur between sessions. In order to test the stability of the proposed system in real-world usage conditions, data were collected in two separate sessions, morning and evening, and the CORAL-based domain adaptation method was applied to reduce the distribution differences between sessions. Thus, the distribution differences that occurred between sessions were statistically balanced, and the need for recalibration of the system was eliminated. In this context, an approach closer to real-world applications is presented by evaluating the time-varying cognitive and physiological states of system users. In addition, by increasing the number of participants, it was shown that the developed system can exhibit similar performance on different individuals, which significantly strengthened the generalizability of the system. When participant diversity was evaluated together with the consistent results obtained despite biological and cognitive variations, it was revealed that the system is applicable not only for certain individuals but also for wider user groups. In addition, by showing all moving object trajectories to the user on a single screen during the data recording phase, it was aimed to integrate the system more easily into daily life. This holistic stimulus presentation both increased user ergonomics and simplified the system installation process, providing its suitability for practical applications. Another particularly striking point is that, while the same number of channels (four) were used in the classification process, the system was tested using fewer features (four) and recording less data. This situation is advantageous especially for low-resource portable systems and makes the system applicable even under hardware limitations.

Despite the promising results of the designed system, there are some limitations to this study. For example, in the designed system, the electrode positions were positioned to be exactly the same in both sessions in order to minimize the distribution differences between sessions during the data recording phase. To eliminate the impedance difference, the electrodes were wetted with salt water in each session. Additionally, the system provides intersession stability for a single user. Existing trained models provided high accuracy and ITR rate for the same person. However, the model trained for one user was not examined in the study given by the other user. Additionally, if there is deviation in electrode positions, system performance may be negatively affected. Another problem is that the system was not tested in real time. The system, which is intended to provide maximum suitability for real-time applications, must be tested in real time. The problem observed during the data recording phase is that the Emotiv Flex EEG headset used creates a feeling of pain in the users due to pressure in the following minutes. Additionally, the device cannot be adjusted according to head size. In this study, which aimed to highlight the concept of comfort, it was seen that the EEG headset caused problems, especially for people with large head sizes. In this study, three different machine learning algorithms were used in the classification stage, and the results were compared. However, testing the proposed method with deep learning methods can increase system performance. Moreover, although studies have suggested that movement trajectories are intuitive and comfortable, more studies are needed to evaluate the cognitive workload and fatigue caused by these tasks. Thanks to the improvements identified and proposed solutions, the designed hybrid BCI system has the potential to turn into a more comfortable, real-life, and comfortable system.

## 6. Conclusions

In this study, in order to overcome the basic limitations of EEG-based systems in user comfort, intersession instability, reliability, and system activation, a two-stage hybrid BCI system is proposed, where EOG artefacts occurring in EEG signals and the SSVEP response caused by the 7 Hz LED are used, and both time and frequency domain qualities are used together. In this system, the user can safely activate the interface using a single low-frequency (7 Hz) LED stimulus, which remains outside the 15–25 Hz range associated with increased risk of photosensitive epileptic seizures. Subsequently, four-way object tracking is performed using eye movements derived from EOG artefacts. This structure provides both a security layer that prevents involuntary commands and the opportunity to interact with low cognitive load. The data was collected in two separate sessions, and the stability of the system was tested at the intersession level. The CORAL method applied within the scope of domain adaptation reduced the statistical differences between sessions and enabled the system to operate without the need for recalibration. In the first classification stage of the study, the signals were filtered in the range of 1–15 Hz, and then proportional trapezoidal features were extracted by performing PSD analysis. Feature data was classified by Bagging, SVM, and RF algorithms and accuracy rates of 99.12%, 98.63%, and 98.67% were obtained, respectively.

In the second classification stage, power, energy, and curve fitting (20th degree) features were extracted from the data of movement trajectories. A comparative analysis of polynomial orders was conducted to mitigate the risk of overfitting. It revealed that model performance was highly dependent on the order of the polynomial used for signal fitting (20th-order). Low-order polynomial coefficients (15th-order) underfitted the data, achieving an average accuracy of 74.10%, while high-order polynomial coefficients (25th-order) slightly overfitted the data, reducing overall performance to 80.80% despite local improvements. The 20th-order polynomial achieved the best balance between bias and variance, achieving an average accuracy of 81.95% and the highest class-specific accuracy (87.80% for Class-4). These results confirm that this order provides sufficient model flexibility to capture task-related variation without overfitting noise components. Consequently, the 20th-order polynomial was determined to be the optimal configuration, effectively improving classification accuracy and signal interpretability while preventing overfitting. The extracted features (20th-order) were classified with RF, Bagging, and SVM algorithms and average accuracy rates of 79.87%, 81.54%, and 76.31% were obtained, respectively. The classification process was then repeated by applying the CORAL method to the data. An average accuracy rate of 93.80%, 37.54 (bits/min) ITR value, and 94.07% precision were obtained for the RF algorithm, and an average accuracy rate of 92.02%, 35.38 (bits/min) ITR value, and 92.82% precision were obtained for the SVM algorithm. The Bagging algorithm showed the highest performance with 94.29% accuracy, 38.35 (bits/min) ITR, 94.55% precision, and 94.42% F1-measure. The Bagging algorithm with the CORAL domain adaptation method achieved the best performance between sessions, reaching an accuracy rate of 94.29% from an average accuracy rate of 81.54%, despite individual variations. An ANOVA analysis was performed to evaluate system performance based on the extracted features and whether reducing the feature matrix would maintain model robustness. Initially, 92 features (23 × 4 per channel) were extracted from the EEG data. After applying a one-way ANOVA F-test with a significance threshold of *p* < 0.05, 66 features were determined to be statistically significant and retained for classification. When the classifiers were retrained using the reduced feature set, only a small decrease in performance was observed compared to the results obtained with the full 92 features. Average accuracies decreased from 93.80% to 90.93% for the RF algorithm, from 92.02% to 90.11% for the SVM, and from 94.29% to 92.09% for the Bagging classifier. Despite this small reduction, all models maintained high and consistent levels of accuracy, confirming that the removed features contributed little to class separation. The Bagging classifier again achieved the highest accuracy (92.09%), demonstrating that the subset selected by ANOVA retained the most discriminative and meaningful features. Furthermore, by removing redundant and correlated components, the classification became more robust to noise and reduced the tendency for overfitting. Overall, the results demonstrated that ANOVA-based feature selection could successfully reduce the feature space by approximately 30% (from 92 to 66) while maintaining high classification performance. This confirmed that the proposed hybrid BCI framework effectively captures essential signal features, and its performance remains robust, interpretable, and reproducible even under reduced feature conditions.

To assess the temporal robustness of the proposed hybrid BCI system, it was quantitatively evaluated by comparing the classification accuracies obtained from two independent recording sessions (morning and evening). The Bagging classifier trained on the morning dataset was then tested on both morning and evening data to examine the stability of performance under session variability. A Wilcoxon signed-rank test was applied to the paired-class accuracies obtained from the two sessions. The test yielded a *p*-value of 0.125, which is greater than the significance threshold (*p* < 0.05). This result confirmed that there was no statistically significant difference between the classification accuracies obtained from the morning and evening sessions. The proposed hybrid BCI system demonstrated temporal stability and strong generalization, maintaining high accuracy despite diurnal variations and session-to-session non-stationarity. These findings support the robustness of the system for practical BCI applications where recording conditions and user situations may change over time. In addition, the comfort-oriented selection of visual stimuli used in the system and the establishment of a careful movement-based structure during the direction determination phase have provided an effective solution for problems such as visual fatigue. Although user experience remains in the background in traditional systems, the proposed system offers a holistic approach that prioritizes both technical success and user ergonomics. As a result, the proposed hybrid BCI system with its low channel count, intersession stability, security structure that prevents unintentional commands, and relatively high classification performance, is promising in the transition from the laboratory environment to real-world applications.

## Figures and Tables

**Table 1:** Accuracy rates of the first classification stage where the system detects LED activation.

Classifiers	RF			SVM			Bagging		
Classes	Class-1	Class-2	Acc.	Class-1	Class-2	Acc.	Class-1	Class-2	Acc.
Sub-1	99.00	92.00	97.60	99.05	92.00	97.64	99.80	96.60	99.16
Sub-2	99.55	97.60	99.16	99.15	92.00	97.72	99.90	97.80	99.48
Sub-3	99.20	92.00	97.76	99.65	96.80	99.08	99.70	97.80	99.32
Sub-4	99.85	97.40	99.36	99.60	97.80	99.24	99.75	97.20	99.24
Sub-5	99.70	97.80	99.32	99.50	97.60	99.12	99.85	97.40	99.36
Sub-6	99.85	96.60	99.20	99.15	92.00	97.72	99.85	96.80	99.24
Sub-7	99.10	92.00	97.68	99.70	97.00	99.16	99.50	95.20	98.64
Sub-8	99.05	92.00	97.64	99.00	92.00	97.60	100.0	95.80	99.16
Sub-9	99.00	92.00	97.60	99.35	98.00	99.08	99.90	97.60	99.44
Sub-10	99.75	98.00	99.40	99.15	92.00	99.72	99.50	97.80	99.16
Sub-11	99.80	97.80	99.40	99.70	97.40	99.24	100.0	95.60	99.12
Sub-12	99.80	96.60	99.16	99.00	92.00	97.60	99.05	92.00	97.64
Sub-13	99.30	97.40	98.92	99.95	98.80	99.72	100.0	95.60	99.12
Sub-14	99.50	95.20	98.64	99.15	92.00	97.72	99.70	97.00	99.70
Sub-15	99.70	97.80	99.32	99.70	97.00	99.16	99.80	96.60	99.16
Avg. (%)	99.47	95.48	98.67	99.38	94.96	98.63	99.75	96.45	99.12

**Table 2:** Accuracy rates of the classification of feature data generated using the 15th, 20th, and 25th polynomial degrees with the Bagging algorithm.

Classes	n = 15	n = 20	n = 25
Class-1	70.40	80.20	74.00
Class-2	76.80	79.60	86.40
Class-3	72.40	81.20	78.00
Class-4	76.80	87.80	84.80
Avg. (%)	74.10	81.95	80.80

**Table 3:** Accuracy, ITR, precision, recall, and F1-measure values of the data to which the CORAL adaptation method was applied.

Classifier	Classes	Sub-1	Sub-2	Sub-3	Sub-4	Sub-5	Sub-6	Sub-7	Sub-8	Sub-9	Sub-10	Sub-11	Sub-12	Sub-13	Sub-14	Sub-15	Avg. (%)
RF	Class-1	82.20	97.60	91.60	93.40	86.80	98.20	76.80	85.20	90.80	93.40	80.40	89.80	93.20	92.40	91.80	89.57
	Class-2	93.80	94.80	96.00	95.80	91.60	99.00	95.20	96.80	95.80	99.80	93.40	95.80	95.40	97.20	95.20	95.70
	Class-3	82.80	96.60	94.60	93.00	96.20	100.0	92.00	91.80	96.40	95.60	92.00	90.80	94.40	91.80	92.60	93.37
	Class-4	98.00	98.00	96.60	96.00	96.20	99.60	94.60	98.80	96.00	97.80	94.60	95.80	95.80	95.60	94.60	96.53
	Acc. (%)	89.20	96.75	94.70	94.55	92.70	99.20	89.65	93.15	94.75	96.65	90.10	93.05	94.70	94.25	93.55	93.80
	ITR (bit/min)	32.33	41.06	38.45	38.20	36.09	45.56	32.68	36.60	38.42	40.76	33.17	36.44	38.42	37.90	37.00	37.54
	Precision	89.45	96.78	94.97	94.76	93.18	99.23	90.10	93.35	94.87	97.01	90.59	93.37	94.90	94.59	93.90	94.07
	Recall	89.20	96.75	94.70	94.55	92.70	99.20	89.65	93.15	94.75	96.65	90.10	93.05	94.70	94.25	93.55	93.80
	F1-Measure	89.32	96.76	94.83	94.65	92.94	99.21	89.87	93.25	94.81	96.83	90.34	93.21	94.80	94.42	93.72	93.93
SVM	Class-1	77.20	97.80	86.20	88.20	88.60	98.80	82.60	82.60	88.40	86.20	81.20	87.80	86.80	87.00	88.40	87.18
	Class-2	94.20	92.20	95.20	97.40	86.20	96.40	95.60	98.40	94.60	94.60	93.80	92.00	90.80	96.00	95.00	94.16
	Class-3	81.60	89.40	89.40	84.80	85.20	97.80	81.20	90.40	89.20	91.40	81.00	89.20	90.40	92.40	87.60	88.06
	Class-4	99.40	97.00	100.0	99.80	99.60	98.80	98.40	98.00	98.80	100.0	99.00	99.80	100.0	93.40	98.00	98.66
	Acc. (%)	88.10	94.10	92.70	92.55	89.90	97.95	89.45	92.35	92.75	93.05	88.75	92.20	92.00	92.20	92.25	92.02
	ITR (bit/min)	31.16	37.53	36.05	35.90	33.10	42.77	32.49	35.78	36.06	36.47	32.01	35.41	35.20	35.42	35.48	35.38
	Precision	88.30	94.53	93.01	92.75	91.45	98.01	90.21	92.61	93.18	93.42	89.60	92.70	92.90	96.71	92.96	92.82
	Recall	88.10	94.10	92.70	92.55	89.90	97.95	89.45	92.35	92.75	93.05	88.75	92.20	92.00	92.20	92.25	92.02
	F1-Measure	88.20	94.31	92.85	92.65	90.67	97.98	89.83	92.48	92.96	93.23	89.17	92.45	92.45	94.40	92.60	92.41
Bagging	Class-1	82.80	98.00	93.40	92.40	84.80	99.80	77.20	83.00	94.20	92.40	78.00	94.00	93.00	93.00	94.00	90.01
	Class-2	96.60	95.20	98.20	98.20	91.00	100.0	95.60	95.20	98.00	97.60	95.40	97.80	98.20	98.20	97.40	96.84
	Class-3	83.60	97.60	96.80	97.00	94.80	100.0	93.20	86.90	97.40	97.20	95.00	96.40	96.00	96.20	97.00	95.00
	Class-4	94.20	98.00	94.00	94.80	95.00	100.0	94.00	99.90	94.20	94.80	94.00	94.60	94.40	94.00	94.20	95.34
	Acc. (%)	89.30	97.20	95.60	95.60	91.40	99.95	90.00	91.25	95.95	95.50	90.60	95.70	95.40	95.35	95.65	94.29
	ITR (bit/min)	32.46	41.75	39.48	39.52	34.54	49.53	33.06	34.38	39.93	39.35	33.71	39.63	39.23	39.17	39.56	38.35
	Precision	89.67	97.21	95.89	95.85	91.81	99.95	90.39	91.55	96.20	95.77	90.90	95.96	95.67	95.61	95.89	94.55
	Recall	89.30	97.20	95.60	95.60	91.40	99.95	90.00	91.25	95.95	95.50	90.60	95.70	95.40	95.35	95.65	94.29
	F1-Measure	89.48	97.20	95.74	95.72	91.60	99.95	90.19	91.40	96.07	95.63	90.75	95.83	95.53	95.48	95.77	94.42

**Table 4:** Accuracy and ITR values obtained with 66 features selected as a result of applying the ANOVA feature selection method to the data adapted with CORAL.

Classifier	Classes	Sub-1	Sub-2	Sub-3	Sub-4	Sub-5	Sub-6	Sub-7	Sub-8	Sub-9	Sub-10	Sub-11	Sub-12	Sub-13	Sub-14	Sub-15	Avg. (%)
RF	Class-1	89.00	94.15	91.20	93.20	91.60	92.20	89.20	92.20	92.80	94.60	88.20	90.80	92.20	91.40	91.20	91.69
	Class-2	85.20	92.25	88.80	90.55	89.20	91.80	87.20	89.80	89.00	93.00	86.60	88.50	87.40	90.40	90.80	89.22
	Class-3	84.80	90.60	86.40	87.60	86.40	91.20	85.60	87.35	90.00	90.20	85.60	86.50	88.10	88.60	91.40	87.89
	Class-4	91.80	97.00	92.00	95.65	94.40	94.40	93.00	95.05	94.80	98.00	92.00	95.60	95.50	94.80	96.00	94.83
	Acc. (%)	87.70	93.50	90.10	91.70	90.30	92.40	88.75	91.10	91.65	93.95	88.10	90.35	90.80	91.30	92.35	90.93
	ITR (bit/min)	30.76	36.89	33.16	34.86	33.37	35.64	31.79	34.21	34.81	37.43	31.14	33.42	33.89	34.43	35.58	34.03
SVM	Class-1	87.30	93.15	92.00	89.50	89.00	93.85	90.40	91.40	91.00	92.25	87.60	92.60	90.05	92.55	92.80	91.03
	Class-2	85.20	91.00	88.00	88.70	88.40	92.40	87.60	89.20	88.40	90.35	84.20	89.00	89.20	90.00	87.60	88.61
	Class-3	83.50	89.25	88.60	86.80	85.40	90.20	85.60	89.00	86.80	87.20	84.00	88.60	84.35	89.25	88.00	87.10
	Class-4	90.80	96.00	95.00	93.00	92.00	96.95	93.00	95.00	94.00	95.00	90.00	96.00	92.80	94.60	95.00	93.94
	Acc. (%)	86.70	91.85	90.90	89.05	88.70	93.35	89.15	91.15	90.05	91.20	86.45	91.55	89.10	91.60	90.85	90.11
	ITR (bit/min)	29.80	35.02	34.00	32.09	31.74	36.72	32.19	34.26	33.11	34.32	29.57	34.70	32.14	34.75	33.94	33.22
Bagging	Class-1	88.40	93.60	95.80	92.60	91.20	97.40	89.80	92.00	93.80	93.00	89.80	97.00	94.00	93.20	95.60	93.14
	Class-2	84.60	92.40	93.60	91.40	87.80	95.20	86.20	90.00	90.00	90.80	85.80	92.20	91.40	90.60	92.00	90.26
	Class-3	83.80	90.20	91.60	88.40	88.40	93.80	84.20	87.80	91.20	88.00	85.60	91.00	89.60	89.20	91.00	88.92
	Class-4	91.00	97.80	98.00	96.00	95.00	99.60	92.00	95.00	96.00	96.00	93.00	99.00	97.00	96.00	99.00	96.02
	Acc. (%)	86.95	93.50	94.75	92.10	90.60	96.50	88.05	91.20	92.75	91.95	88.55	94.80	93.00	92.25	94.40	92.09
	ITR (bit/min)	30.04	36.89	38.40	35.30	33.68	40.66	31.10	34.32	36.03	35.13	31.59	38.46	36.32	35.47	37.97	35.42

**Table 5:** Wilcoxon *p* value of accuracy rates obtained from training and test data using the Bagging algorithm.

Classes	Morning Trials (%)	Evening Trials (%)	*p* Value
Class-1	97.40	93.14	*p* = 0.125
Class-2	93.80	90.26	
Class-3	92.80	88.92	
Class-4	100.0	96.02	
Overall (%)	96.00	92.09	

**Table 6:** Comparison of the current study with and without CORAL applied with studies conducted in the field.

Reference	Number of Participants	Device	Signal Type	Feature Extraction	Classifier/Algorithm	Performance Metrics
[27]	10	8-ch custom EEG amplifier	EEG (SSVEP) + EOG	CCA and FBCCA for SSVEP; raw EOG waveform	CCA/FBCCA + decision fusion	Acc. 94.75%; ITR 108.63 (bits/min)
[57]	12	8-ch EEG + EOG electrodes	EEG (SSVEP) + EOG	Task-prior probability (SSVEP); saccade detection (EOG)	Bayesian update of target dist.	Acc. 81.67%
[58]	10	8-ch EEG + EOG (IIT-Madras setup)	EEG (SSVEP) + EOG	Two-stage: Eye gestures (blinks/winks) for group; PSD for SSVEP targets	Threshold detection + CCA	Acc. 94.16%; ITR 70.99 (bits/min)
[15]	15	9-electrode EEG	EEG (SSVEP) + EOG	Bandpass 0.1–15 Hz; diff. features (EOG); Filter-Bank CCA (EEG)	Multi-threshold EOG; CCA/FBCCA	Cue-based Acc. 92.09%; ITR 35.98 (bits/min)
[12]	22	9-electrode EEG + EOG	EEG (MI) + EOG (blinks/brow)	CSP + adaptive thresholds (EEG); blink waveform features (EOG)	LDA-based MI classifier; threshold (EOG)	5 subjects achieved > 80% MI Acc.
[59]	12	21-ch EEG cap (visual cortex)	EEG (SSVEP)	Bandpass 3–40 Hz; CCA and MSI (no training)	CCA (with MSI comparison)	Screen: Acc. 90.7%, ITR 33.8 (bits/min), Goggles: Acc. 98.8%, ITR 44.1 (bits/min).
[60]	7	1-ch EEG (forehead)	EEG (SSVEP)	Time domain samples (0.5–2 s epochs)	CNN and LSTM (deep learning)	Acc. 90.0%
[24]	10	4-ch dry EEG	SSVEP + EOG	PSD	RF	Acc. 97.89%; ITR 36.75 (bits/min)
[26]	5	4-ch EOG + eye-tracker + force fb.	EEG (SSVEP) + EOG + others	Threshold triggers (EOG blinks); eye gaze tracking; force feedback loop	Threshold logic + manual control	Acc. 93.0%
Our Work (without CORAL)	15	4-ch EEG	EEG(SSVEP) + EOG artefact	PSD for SSVEP; energy, power, and polynomial curve fitting for EOG	RF, SVM, Bagging	Acc. 81.54%, ITR 25.25 (bits/min)
Our Work (with CORAL)	15	4-ch EEG	EEG(SSVEP) + EOG artefact	PSD for SSVEP; energy, power, and polynomial curve fitting for EOG	RF, SVM, Bagging	Acc. 94.29%, ITR 38.35 (bits/min)

## Data Availability

The raw data supporting the conclusions of this article will be made available by the authors on request.

## Abbreviations

The following abbreviations are used in this manuscript:

Acc	Accuracy
Avg	Average
Bagging	Bootstrap Aggregating
BCI	Brain–Computer Interface
CCA	Canonical Correlation Analysis
CORAL	Correlation Alignment
EEG	Electroencephalography
EOG	Electrooculography
FBCCA	Filter-Bank Canonical Correlation Analysis
ITR	Information Transfer Rate
LDA	Linear Discriminant Analysis
LED	Light-Emitting Diode
MI	Motor Imagery
PSD	Power Spectral Density
RF	Random Forest
SSVEP	Steady-State Visual Evoked Potential
Sub	Subject
SVM	Support Vector Machine
VEP	Visual Evoked Potential
